# Serum metabolites associated with wholegrain consumption using nontargeted metabolic profiling: a discovery and reproducibility study

**DOI:** 10.1007/s00394-022-03010-x

**Published:** 2022-10-06

**Authors:** Stefania Noerman, Jyrki K. Virtanen, Marko Lehtonen, Carl Brunius, Kati Hanhineva

**Affiliations:** 1 Present Address: Division of Food and Nutrition Science, Department of Biology and Biological Engineering, Chalmers University of Technology, Gothenburg, Sweden; 2 Present Address: Department of Life Technologies, Food Chemistry and Food Development Unit, University of Turku, Turku, Finland; 3 Institute of Public Health and Clinical Nutrition, University of Eastern Finland, Kuopio, Finland; 4 School of Pharmacy, University of Eastern Finland, Kuopio, Finland

**Keywords:** Nontargeted metabolomics, Wholegrain, LC–MS, Dietary assessment, Biomarker

## Abstract

**Purpose:**

To identify fasting serum metabolites associated with WG intake in a free-living population adjusted for potential confounders.

**Methods:**

We selected fasting serum samples at baseline from a subset (*n* = 364) of the prospective population-based Kuopio Ischaemic Heart Disease Risk Factor Study (KIHD) cohort. The samples were analyzed using nontargeted metabolomics with liquid chromatography coupled with mass spectrometry (LC–MS). Association with WG intake was investigated using both random forest followed by linear regression adjusted for age, BMI, smoking, physical activity, energy and alcohol consumption, and partial Spearman correlation adjusted for the same covariates. Features selected by any of these models were shortlisted for annotation. We then checked if we could replicate the findings in an independent subset from the same cohort (*n* = 200).

**Results:**

Direct associations were observed between WG intake and pipecolic acid betaine, tetradecanedioic acid, four glucuronidated alkylresorcinols (ARs), and an unknown metabolite both in discovery and replication cohorts. The associations remained significant (FDR<0.05) even after adjustment for the confounders in both cohorts. Sinapyl alcohol was positively correlated with WG intake in both cohorts after adjustment for the confounders but not in linear models in the replication cohort. Some microbial metabolites, such as indolepropionic acid, were positively correlated with WG intake in the discovery cohort, but the correlations were not replicated in the replication cohort.

**Conclusions:**

The identified associations between WG intake and the seven metabolites after adjusting for confounders in both discovery and replication cohorts suggest the potential of these metabolites as robust biomarkers of WG consumption.

**Supplementary Information:**

The online version contains supplementary material available at 10.1007/s00394-022-03010-x.

## Introduction

Consumption of wholegrain (WG) cereals has been shown to convey various health benefits, such as lower inflammation markers [1] as well as reduced risk of type 2 diabetes [2], cardiovascular diseases, and colorectal and prostate cancer [3]. Fiber and phytochemical content have been suggested as the key components responsible for health benefits via modulation of, e.g., postprandial glycemic response and lowering serum LDL cholesterol [4, 5]. In addition, the fiber content could potentially influence the gut microbial community [6], which may induce changes in the microbial metabolites and metabolic outcomes thereafter. To advance our understanding of the mechanisms by which WG influence health outcomes, dietary assessment is crucial. However, subjective reporting of dietary intake is prone to misreporting due to, e.g., recall bias, error in estimation of portion size, or giving favorable or socially desirable answers. The application of both subjective reporting and objective measurement of biomarkers can provide complementary estimation of dietary intake, which may not be achievable using only either one of the approaches.

The FoodBall consortium has classified dietary biomarkers as indicators to reflect (1) the consumption of food, its compounds or components, or part of a dietary pattern, or (2) the effect or implicated physiological and health status [7]. In the case of WG, odd-chain alkylresorcinols (ARs) and their homologues have been widely explored as intake biomarkers of WG rye and wheat, while the even-chain ones seem to be specific for quinoa [5, 8, 9]. More recently, trimethylamine-N-oxide and various betainized compounds have been reported from consuming a WG-rich diet [10, 11]. In addition, lower levels of several endogenous compounds, such as serotonin, taurine, and glycerophosphocholine, and phosphatidylcholines (PCs) have also been reported after WG intake [12, 13]. However, the metabolism of these compounds in the body seems to depend on individual factors, such as age, sex, and BMI [14, 15]. In addition, many factors covary with habitual WG intakes, such as higher physical activity, lower tendency to smoke, and lower alcohol consumption [16]. On top of that, the risk of non- or low compliance in the intervention studies [17] may make it more complicated to disentangle the effect of individual factors on WG-associated metabolites. Hence, there is a need to establish a panel of diet-derived and/or endogenous metabolites associated with WG intake independent of confounding factors in free-living populations.

Applications of nontargeted metabolomics in health sciences have been shown to reflect the contribution of intrinsic and (semi-) modifiable factors, including genetics [18], endogenous metabolic pathways, and gut microbiota [19], as well as lifestyle factors, such as diet [20], stress [21], and other environmental exposures [22]. Profiling the blood metabolome may hence provide information about lifestyle, environmental exposure, and other information about the individuals, including biological mechanisms underlying the relationship between nutrition and health [22–24].

Here we present the application of nontargeted metabolic profiling to assess blood metabolites associated with WG consumption in a prospective population-based cohort study. Based on the presumed causal relationship between WG intake and the blood metabolome, associations were adjusted for confounders (age, BMI, smoking, physical activity, energy and alcohol consumption). Finally, the discovered metabolites were checked if they could be replicated in an independent subset.

## Materials and methods

### Study population

The samples for this study were obtained from the Finnish middle-aged male participants of the Kuopio Ischaemic Heart Disease Risk Factor Study (KIHD). KIHD is an ongoing population-based prospective cohort study in Eastern Finland [25]. The baseline examination took place in 1984–1989. 2682 men aged 42–60 years (83% of those who were eligible) participated in the baseline examinations.

#### Dietary assessment

Participants self-reported their dietary intake at baseline using a 4-day food record [26]. To ensure reporting accuracy, the participants received instructions on how to fill out the food record and a picture book containing a list of 126 foods and drinks typically consumed in Finland during the 1980s. Each item included a corresponding estimation of portion size based on household measures to ensure proper assessment and recording [27]. During a study visit, a nutritionist checked the completed food records with the participant to improve accuracy [25].

The definition of WG followed the definition by the HEALTHGRAIN project [28], including downstream products, such as pasta. The KIHD database does not include information on intakes of individual grains. In the mid-to-late 1980s in Finland, wheat and rye were the most commonly consumed grains, followed by oat, rice, and barley [29]. However, in the KIHD cohort, WG pasta or rice intake was very uncommon (Table 1). The calculation of food and nutrient intakes was performed using the NUTRICA^®^ 2.5 software (Social Insurance Institution, Turku, Finland), based mainly on the Finnish database of the nutrient composition of foods.

**Table 1:** Baseline characteristics and dietary intake of study participants in each subset

	Study cohorts^a^					
	Discovery cohort (DC)		Replication cohort (RC)		Total	
	*n*	Median (IQR)	*n*	Median (IQR)	*n*	Median (IQR)
Age, year	364	54.33 (48.50, 54.50)	239	54.33 (48.62, 54.50)	564	54.33 (48.56, 54.50)
Body mass index, kg/m^2^	362	25.90 (24.31, 28.29)	239	26.47 (24.81, 27.98)	562	26.16 (24.53, 28.12)
Waist-to-hip ratio	305	0.94 (0.91, 0.98)	199	0.94 (0.91, 0.97)	470	0.94 (0.91, 0.98)
Leisure-time physical activity, kcal/day	363	83.63 (24.66, 193.26)	239	77.34 (34.55, 179.98)	563	78.66 (28.79, 181.52)
Current smoker/past smoker, %	364	34.3/29.9	239	1.5/38.5	564	22.7/33.0
Cigarette packs/year	357	0.00 (0.00, 13.50)	239	0.00 (0.00, 0.00)	557	0.00 (0.00, 0.00)
Alcohol consumption, g/week	363	27.15 (5.95, 88.00)	239	12.00 (1.78, 37.02)	563	21.80 (3.80, 63.28)
Fasting serum insulin, mU/L	360	9.30 (7.00, 12.33)	235	10.30 (7.60, 13.30)	556	9.80 (7.30, 12.72)
Blood glucose, mmol/L	364	4.50 (4.30, 4.80)	238	4.60 (4.30, 4.90)	563	4.60 (4.30, 4.90)
Serum total cholesterol, mmol/L	363	5.99 (5.25, 6.68)	236	5.62 (5.04, 6.38)	560	5.84 (5.14, 6.57)
Serum VLDL cholesterol, mmol/L	361	0.48 (0.28, 0.77)	236	0.43 (0.30, 0.65)	558	0.47 (0.29, 0.72)
Serum LDL cholesterol, mmol/L	361	4.12 (3.48, 4.90)	236	3.78 (3.20, 4.56)	558	3.94 (3.35, 4.78)
Serum HDL cholesterol, mmol/L	362	1.28 (1.08, 1.47)	236	1.29 (1.13, 1.52)	559	1.27 (1.10, 1.48)
Serum triglycerides, mmol/L	357	1.08 (0.76, 1.47)	237	1.03 (0.74, 1.41)	555	1.05 (0.76, 1.45)
Serum C-reactive protein, mg/L	364	1.08 (0.65, 2.13)	239	0.90 (0.53, 1.75)	564	1.04 (0.63, 2.00)
Mean systolic blood pressure (mmHg)	362	131.92 (123.54, 143.92)	239	131.67 (122.83, 141.33)	562	131.92 (123.33, 142.33)
Mean diastolic blood pressure (mmHg)	362	88.33 (82.00, 95.67)	238	89.67 (83.17, 95.63)	561	89.00 (82.33, 95.67)
Dietary components						
Fruits and berries, g/day^b^	364	113.99 (38.54, 216.69)	239	126.75 (61.10, 209.18)	564	118.99 (44.61, 207.79)
Vegetables, g/d^c^	364	103.57 (59.36, 158.83)	239	104.58 (66.83, 159.23)	564	102.70 (60.45, 157.06)
Total grain products, g/d	364	255.41 (194.00, 312.80)	239	265.90 (212.35, 315.06)	564	258.11 (204.84, 314.10)
Total whole grains, g/d	364	155.28 (112.48, 208.83)	239	156.38 (120.53, 206.55)	564	154.90 (115.54, 207.29)
Whole grains, excluding rice and pasta, g/d	364	155.28 (112.48, 208.83)	239	156.38 (120.53, 206.55)	564	154.90 (115.54, 207.29)
Refined grains, g/d	364	87.72 (59.86, 121.55)	239	98.65 (75.28, 129.65)	564	90.95 (65.69, 125.74)
Nutrients						
Energy (kcal/d)	364	2,445.39 (2,065.45, 2,787.99)	239	2,454.97 (2,174.06, 2,842.69)	564	2,452.41 (2,123.48, 2,803.00)
Carbohydrate, %E	364	43.33 (38.70, 48.56)	239	44.11 (40.83, 48.28)	564	43.65 (39.67, 48.33)
Protein, %E	364	15.19 (14.15, 16.82)	239	15.17 (13.86, 16.76)	564	15.18 (13.97, 16.82)
Total fat, %E	364	38.48 (34.15, 42.77)	239	38.77 (35.34, 42.30)	564	38.54 (34.56, 42.34)
SFA, %E	364	17.95 (14.81, 21.03)	239	18.48 (15.59, 21.21)	564	18.17 (15.27, 21.10)
MUFA, %E	364	11.65 (10.22, 12.87)	239	11.61 (10.12, 12.99)	564	11.64 (10.15, 12.99)
PUFA, %E	364	4.21 (3.43, 5.23)	239	4.36 (3.44, 5.33)	564	4.22 (3.44, 5.27)
Trans fatty acids, %E	364	1.00 (0.81, 1.22)	239	1.03 (0.87, 1.24)	564	1.02 (0.84, 1.23)
Fiber, g/d, energy adjusted	364	24.52 (19.54, 30.36)	239	25.35 (20.87, 29.74)	564	24.83 (20.26, 30.17)
Cholesterol, mg/d, energy adjusted	364	387.11 (328.15, 458.82)	239	382.65 (328.82, 462.50)	564	385.71 (328.68, 460.28)

All values are presented in median ± interquartile range (IQR), except for proportion of current and past smokers. Dietary data are presented in 4-day-food-record median ± interquartile range (IQR)

%*E* percentage of energy intake, *SFA* saturated fatty acids, *MUFA* monounsaturated fatty acids, *PUFA* polyunsaturated fatty acids

^a^*DC* discovery cohort [30], *RC* replication cohort [31]

^b^Excluding jams and juices

^c^Excluding potatoes and vegetable juices

#### Selection of samples

Serum samples and data for this study were taken from two independent subsets within the KIHD cohort. The discovery cohort (DC) was selected from a previous study on adherence to a healthy Nordic diet and incidence of coronary artery disease within a mean follow-up of 20.4 years (*n*_DC_ = 364) [30]. The replication cohort (RC) was taken from a study investigating the association between egg consumption and the incidence of type 2 diabetes after a mean follow-up of 19.3 years [31]. From the original number of participants (*n* = 239), 39 participants were excluded, since they were already included in the DC (*n*_RC_ = 200).

#### Collection of blood samples and other measurements

Blood samples were collected during the baseline examination visits in 1984–1989. Participants were instructed to abstain from alcohol consumption for 3 days and from smoking and eating for 12 h before examination visits between 08.00 and 10.00 on Tuesdays–Thursdays [32]. After 30-min rest in supine position, venous blood samples were drawn without a tourniquet [32]. Serum was separated by centrifugation at 2000*g* for 10 min (20 °C) after coagulation at room temperature for an hour [32]. The obtained serum samples were stored at − 80 °C until LC–MS analysis in 2016 for RC and 2018 for DC.

Body mass index (BMI) was calculated as body weight (in kg) divided by the square of height (in m^2^). The recording of habitual leisure-time physical activity [33], smoking and alcohol consumption in the past 12 months and measurement of blood pressure [34] have been described previously.

### Metabolomics analysis

Sample randomization and preparation steps have been described in previous publications [30, 31]. After the samples were thawed entirely on ice water for approximately 3 h, 100 µL of each sample was mixed with 400 µL of acetonitrile then pipetted into 96-well plate filter plate layered with 96-well plate. Centrifugation (700*g*, 4 °C, 5 min) was performed to obtain protein-free filtrate [35] which was directly used for LC–MS injection.

Data acquisition for nontargeted metabolic profiling analysis was performed at the LC–MS metabolomics center (Biocenter Kuopio, University of Eastern Finland). Two different LC–MS systems were employed for the DC and RC [30, 31]. The LC systems for the DC and RC were Vanquish UHPLC (Thermo Fischer Scientific) and 1290 Infinity Binary UPLC (Agilent Technologies), respectively. Both systems utilized two chromatographic techniques: reversed-phase (RP) (Zorbax Eclipse XDB C18, 2.1 × 100 mm, 1.8 μm, Agilent Technologies, Palo Alto, CA, USA) and hydrophilic interaction chromatography (HILIC) chromatography (Acquity UPLC^®^ BEH Amide 1.7 µm, 2.1 × 100 mm, Waters Corporation, Milford, MA, USA). The injection volume was 1 µL for each sample. A pooled sample from all biological samples per experiment was injected at the beginning and after every 12 samples throughout LC–MS run for quality control and drift correction.

The MS systems used Q Exactive Focus Orbitrap MS (Thermo Fischer Scientific) for DC and Agilent 6540 Q-TOF (Agilent Technologies) for RC [30, 31], both with high resolution and accuracy. The data were acquired in both positive (ESI+) and negative (ESI−) electrospray ionization modes. At the end of the analysis, data-dependent MS2 were acquired for each mode. Further information about the LC–MS instruments setup and data acquisition parameters can be obtained from the previous publications [30, 31].

#### Discovery cohort

Peak-picking was performed using MS-Dial version 4.20 [36] after converting the raw files to.abf format using Abf Converter. The data were collected with a tolerance of 0.01 Da for MS1 and 0.025 for MS2. Peak detection was performed with a minimum peak height of 10,000 for DC and 1000 for RC due to the different detection units. Preliminary identification was performed in MS-DIAL [36] against the uploaded in-house library with an identification score cutoff of 70% and accurate mass tolerance of 0.015 Da for MS1 and 0.05 for MS2. The tolerance for peak alignment was 0.015 Da and 0.15 min. After alignment, the raw peak area from each mode was then exported to .xlsx files. This data matrix contained 36,584 features from RP−, 30,607 from RP+ , 25,871 from HILIC−, and 15,095 from HILIC+ , which then underwent data preprocessing.

All features were preprocessed using the R package *notame* (https://github.com/antonvsdata/notame) as previously described [21, 35]. The procedures allow correction of drift due to long LC–MS run sequence, missing values imputation, and removal of low-quality signals [35]. Following this procedure, we retained 2829 and 1438 features from HILIC, and 6260 and 6957 features from RP, in ESI + and ESI−, respectively. Thus, the combined data matrix comprised 17,484 features from 364 participants in DC. Before statistical analyses, the peak areas of the features were transformed using log-transformation, followed by normalization by mean-centering and scaling to unit variance.

#### Replication cohort

The metabolomics data of the RC underwent a similar preprocessing procedure as DC described above. One data file from RP+ was corrupted during the peak-picking procedure, so the feature alignment of RP+ was based on 199 samples. The removal of low-quality features yielded 14,110 features from 200 participants in RC, which underwent the same normalization procedures as in DC.

### Statistical analysis

#### Discovery cohort

The selection of features for the identification step employed both multivariate and univariate approaches. Random Forest (RF) using the R package *MUVR* (https://gitlab.com/CarlBrunius/MUVR) that incorporates a repeated double cross-validation scheme was applied to unbiasedly select a set of molecular features ranked based on their importance to predict the total WG intake. Permutation tests (*n* = 40, *p* difference between actual and permutation models  =  1.21e−14) were performed to ascertain that modeling results were not due to overfitting [37]. This variable selection procedure maximized the selection of all relevant features (max model), resulting in a selection of 130 metabolic features. These features were then fitted to a linear regression model (using the built-in *lm* function in R) with WG intake as the independent variable and the normalized metabolite feature as the dependent variable, followed by correction for multiple testing by false discovery rate (FDR). FDR < 0.05 was considered significant.

In addition to the feature selection using random forest, we also performed a partial Spearman correlation test to capture additional features that may not be selected by RF. The correlation test was performed between WG intake and peak area of all features after first regressing both WG intake and peak areas with confounders (age, BMI, leisure-time physical activity, smoking, and intake of alcohol and energy) using the built-in *lm* function. Residuals were then correlated using the built-in *cor.test* function in R. The cutoff of FDR < 0.005 was used to limit the annotation and discussion to a reasonable shortlist of likely relevant metabolites.

#### Replication cohort

143 annotated metabolites in the DC (Supplementary Table 1) were checked if they were also detected in the RC. To estimate the RT of those features in RC, 46 metabolites with confirmed identity based on the mass-to-charge ratio (*m/z*), retention time (RT), and MS2 spectra from both DC and RC were fitted to a locally estimated scatterplot smoothing (LOESS) (Supplementary Table 2) using the built-in *loess* function in R. This number included some metabolites eluting at the range of RT uncovered by the relevant features as anchor points, although they were outside the scope of interest of the current study (Supplementary Table 2). The fitted LOESS was then used to predict (using the built-in *predict* function in R) the RT of the shortlisted features from DC without MS2 spectra in the replication cohort (RC).

Features with *m/z* tolerance of 5 ppm and RT tolerance of 0.5 min from either the RT in the discovery cohort (DC) or LOESS-predicted RT were added to the list of validated metabolites. In total, 61 metabolites with tolerance of mass-to-charge ratio (*m/z*) 5 ppm and retention time (RT) 0.5 min (Supplementary Table 2, Supplementary Methods) were found in the RC. Random forest was not applied to the RC, because RF did not seem to fit the current subset (Q2 = 0.03). The reason could be the selection criteria of the study population which were based on egg intake [31] and were not related to WG intake. Hence, these metabolites were then subjected to the same Spearman correlation and linear regression models as in the DC (Supplementary Table 3, Supplementary Methods).

#### Adjustment for potential confounders

Based on presumed causal relationships depicted in a directed acyclic graph [38] (Supplementary Fig. 1), variables associated with both WG intake as exposure and blood metabolome as outcome were identified as potential confounders. These selected confounders were age, BMI, leisure-time physical activity (kcal/day), smoking (estimated as cigarette packs per day multiplied by years of smoking), and intake of and alcohol (gr/week) and energy (kcal/day). In particular, energy intake was included as a standard multivariate model [39]. These confounders were adjusted for in partial Spearman correlations between WG intake and metabolic features and in adjusted linear models in DC. Both were followed by FDR adjustment. FDR < 0.005 for correlation analysis and FDR < 0.05 for the linear models were considered significant.

The same set of confounders were also adjusted for in the Spearman correlation and linear regression model in the RC, except for smoking, since only one RC participant smoked. FDR < 0.05 for either correlation or linear models was considered significant in the RC. All statistical analyses were performed using R version 4.0.3 [40].

### Compound annotation

**Fig. 1:**
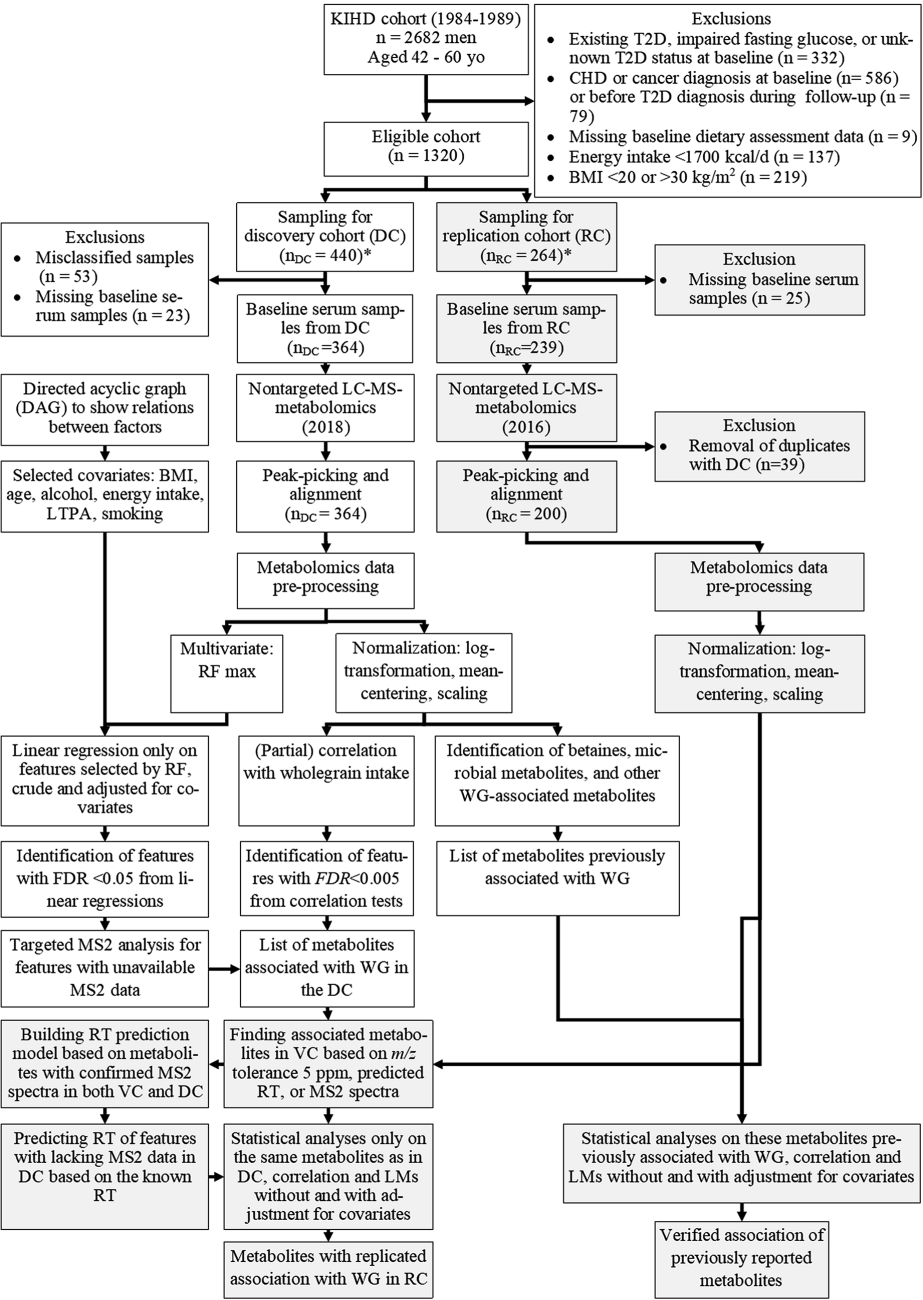
Study flowchart. *BMI* body mass index, *CHD* coronary heart disease, *DAG* directed acyclic graph, *DC* discovery cohort, *FDR* false discovery rate, *KIHD* Kuopio Ischaemic Heart Disease Risk Factor Study, *LC–MS* liquid chromatography–mass spectrometry, *LM* linear regression model, *LTPA* leisure-time physical activity, *MS2* tandem mass spectrometry, *DC* discovery cohort, *RF* max random forest with maximum variable selection, *RT* retention time, *T2D* type 2 diabetes, *RC* replication cohort, *WG* whole grain. *Sample selection criteria have been reported in previous publications according to a healthy Nordic dietary pattern, the incidence of coronary artery disease for DC and egg intake, and incidence of type 2 diabetes for RC [30, 31].

Features in the DC with FDR < 0.05 in linear modeling (*n* = 112) or FDR < 0.005 in correlation analysis (*n* = 245) were added to the shortlist for compound annotation (Fig. 1). The list was further narrowed down by limiting molecular mass < 1000 Da, RT 1–12 min for HILIC and 1–15.5 min for RP modes, leaving 270 features for annotation.

Extracted ion chromatograms and MS2 spectra of differential metabolites were visualized using Freestyle 1.3 (Thermo Fisher Scientific) for annotation purposes. Metabolite annotation was performed based on matching mass, isotopic pattern, and MS2 spectra against existing libraries, either in-house for level I (together with matched RT with pure commercial compound run in the same platform) or online spectral databases (Supplementary Methods) for level II according to the guidelines from the Metabolomics Standard Initiative [41]. The utilized reference libraries for level II identification were MassBank [42, 43], METLIN [44], HMDB version 4.0 [45], and Mass Bank of North America (MoNA). Lipophilic compounds were matched against the in-house library or built-in MS-DIAL library [36] and LIPID MAPS [46]. Phospholipids [47, 48], dihydroxybenzoic acid [49, 50], betaines [51, 52], and alkylresorcinols [12, 49] were annotated based on previously reported MS2 fragments. Features without data-dependent MS2 were subjected to targeted MS2 analysis using the previously described method [30]. Metabolites with compound class annotation based on the fragmentation patterns were reported as level III. Completely unknown compounds with unavailable MS2 data or lacking MS2 interpretation were reported as level IV [41].

### Reproducibility study of metabolites previously associated with wg intake

Besides annotating metabolites from the discovery and replication strategies described above, we further annotated metabolites previously associated with WG intake [10, 12, 13, 49, 52, 53] from the data. This list of metabolites included ARs, betaines, and other metabolites (Supplementary Table 4). In addition, due to the potential interaction between WG, endogenous metabolism, and gut microbiota [54, 55], we also investigated the association between WG intake and some microbial metabolites (Supplementary Table 5) previously reported from gut microbiota or linked to the metabolism of benzoxazinoid or phenolic compounds [54, 56–59].

## Results

Participants' characteristics at baseline and dietary intake data were reported as median (interquartile range (IQR)) (Table 1).

### Metabolites associated with wholegrain intake in the discovery cohort

After removing noise and redundant features or fragments from the same metabolites, 143 metabolites were associated with WG intake based on correlation or linear model after RF variable selection (Supplementary Table 1). Among them, 24 metabolites were directly associated, identified at level I or II (Table 2). Pipecolic acid betaine, aminophenol sulfate, tetradecanedioic acid, dimethoxyphenylpropenoic acid, hydroxyisoleucine, tryptophan, and sinapyl alcohol, were selected in both correlation and RF, followed by a linear model. Three glucuronidated odd-chain ARs were also found in this analysis, namely, AR 19:0-glucuronide, AR 19:1-glucuronide, and AR C21:1-glucuronide (Table 2).

**Table 2:** List of metabolites with level of identification I and II associated with wholegrain intake both in the discovery (DC) and replication cohorts (RC)

Column^a^	ESI	RT (mins)	*m/z*	Adduct type	Metabolite name^b^	ID level^c^	DC								RC							
							Spearman correlation		Partial Spearman correlation		Unadjusted linear model		Adjusted linear model		Spearman correlation		Partial spearman correlation		Unadjusted linear model		Adjusted linear model	
							est	FDR	est	FDR	est	FDR	est	FDR	est	FDR	est	FDR	est	FDR	est	FDR
HILIC	+	3.02	158.118	[M+H] +	Pipecolic acid betaine (B)	I	0.450	**2.6E−15**	0.398	**5.0E−11**	0.006	**1.1E−14**	0.006	**4.8E−11**	0.424	**2.5E−08**	0.328	**7.5E−05**	0.005	**9.1E−07**	0.004	**0.002**
RP	−	11.39	577.376	[M−H] −	AR C21:1-glucuronide	II	0.314	**5.8E−07**	0.286	**3.4E−05**	0.004	**1.8E−07**	0.004	**1.2E−06**	0.324	**5.8E−05**	0.354	**2.1E−05**	0.004	**0.005**	0.004	**0.005**
RP	−	8.63	257.176	[M−H] −	Tetradecanedioic acid	II	0.341	**1.9E−08**	0.281	**5.1E−05**	0.005	**3.7E−10**	0.004	**3.4E−07**	0.272	**1.0E−03**	0.280	**7.3E−04**	0.003	**0.007**	0.004	**0.010**
RP	−	11.02	549.343	[M−H] −	AR C19:1-glucuronide	II	0.396	**9.1E−12**	0.313	**2.4E−06**	0.005	**4.9E−11**	0.005	**4.9E−08**	0.287	**4.8E−04**	0.310	**1.9E−04**	0.003	**0.007**	0.004	**0.010**
RP	−	11.31	551.359	[M−H] −	AR C19:0-glucuronide	II	0.349	**6.3E−09**	0.283	**4.3E−05**	0.005	**3.8E−11**	0.005	**2.6E−08**	0.187	**0.042**	0.278	**7.3E−04**	1.8E**−**03	0.284	0.004	**0.012**
RP	−	11.75	605.407	[M−H] −	AR C23:1-glucuronide	II	0.230	**1.4E−03**	0.273	**9.3E−05**					0.155	0.119	0.256	**2.2E−03**	2.2E**−**03	0.168	0.003	**0.012**
HILIC	+	4.21	148.097	[M+H] +	Hydroxyisoleucine	II	0.231	**1.3E−03**	0.236	**1.2E−03**	0.003	**1.9E−05**	0.004	**1.2E−05**	0.211	**0.021**	0.121	0.190	0.003	**0.028**	2.0E**−**03	0.271
RP	+	6.38	211.096	[M+H] +	Sinapyl alcohol (O)	II	0.290	**6.5E−06**	0.232	**1.6E−03**	0.004	**1.2E−06**	0.003	**2.8E−04**	0.290	**4.7E−04**	0.250	**2.6E−03**	0.003	0.053	2.1E**−**03	0.271
RP	−	2.95	203.003	[M−H] −	Tryptophan	I	0.390	**2.1E−11**	0.307	**4.0E−06**	0.005	**9.5E−11**	0.004	**4.2E−07**	0.129	0.202	0.115	0.196	1.9E**−**03	0.259	1.7E**−**03	0.447
RP	+	5.95	190.086	[M+H] +	3-Indolepropionic acid (M)	I	0.296	**3.6E−06**	0.266	**1.7E−04**					0.028	0.773	− 0.001	0.999	− 7.0E**−**04	0.882	− 1.3E**−**03	0.631
HILIC	+	1.91	160.134	[M+H] +	Valine betaine	I	0.306	**1.3E−06**	0.248	**5.3E−04**					− 0.055	0.608	− 0.106	0.231	− 6.8E**−**04	0.882	− 1.3E**−**03	0.631
RP	+	1.77	247.128	[M+H] +	Gamma-Glu-Val isomer 1	II	0.213	**0.003**	0.127	0.135					− 0.035	0.746	− 0.046	0.667	− 3.2E**−**04	0.942	− 9.3E**−**04	0.881
RP	+	6.73	181.122	[M+H] +	Dihydroactinidiolide	II	0.213	**0.003**	0.126	0.139					0.057	0.592	0.060	0.575	− 3.0E**−**04	0.942	− 6.9E**−**04	0.929
HILIC	+	6.15	147.076	[M+H] +	Glutamine	I	0.238	**7.7E−04**	0.120	0.159					0.111	0.260	0.049	0.646	1.1E**−**03	0.828	3.4E**−**04	0.932
RP	+	11.05	538.387	[M+H] +	LPC(19:0)	II	0.247	**3.8E−04**	0.180	0.026					0.198	**0.034**	0.139	0.135	7.9E**−**04	0.882	4.3E**−**05	0.986
RP	+	13.36	772.583	[M+H] +	PC(17:0/18:2)	II	0.242	**5.7E−04**	0.096	0.260					0.188	**0.042**	0.139	0.135	3.6E**−**04	0.942	− 1.6E**−**04	0.986
RP	−	1.26	188.003	[M−H] −	Aminophenol sulfate	II	0.416	**4.8E−13**	0.341	**1.2E−07**	0.006	**3.2E−14**	0.005	**1.2E−09**								
RP	+	6.85	191.070	[M+H −H_2_O]	Dimethoxyphenylpropenoic acid	II	0.336	**3.8E−08**	0.315	**1.8E−06**	0.005	**1.5E−10**	0.005	**2.7E−08**								
RP	−	3.34	153.020	[M−H] −	Dihydroxybenzoic acid (DHBA) isomer (M)	II	0.294	**4.6E−06**	0.226	**0.002**												
HILIC	+	5.80	261.145	[M+H]+	Gamma-Glu-Leu	II	0.246	**4.1E−04**	0.161	0.055												
RP	+	11.63	580.433	[M+H]+	LPC(22:0)	II	0.230	**1.4E−03**	0.217	0.004												
RP	+	2.10	247.128	[M+H]+	Gamma-Glu-Val isomer 2	II	0.217	**0.003**	0.129	0.129												
HILIC	−	4.22	128.036	[M−H]−	Pyrrolidone carboxylic acid	II	0.217	**0.003**	0.200	0.010												
RP	−	2.09	188.987	[M−H]−	Pyrocatechol sulfate (M)	II	0.213	**0.003**	0.166	0.047												

FDR < 0.005 for correlation analyses and FDR < 0.05 for linear models in DC and FDR < 0.05 for both models in RC were considered significant, printed in bold

Association was assessed by performing Spearman correlation or linear models on features selected by random forest. Both correlation and linear models were adjusted for covariates chosen: age, BMI, alcohol (gr/week) and energy intake (kcal/day), leisure-time physical activity (kcal/d), and smoking (cigarette packs per day x years of smoking). Smoking was excluded from the list of covariates in RC due to insufficient smokers among the participants (proportion of smokers  =  1/200). Est: estimate (rho) of the (partial) Spearman correlation or the linear models; *p* was corrected for multiple comparisons with false discovery rate (FDR). FDR < 0.005 for correlation analyses and FDR < 0.05 for linear models in DC and FDR < 0.05 for both models in RC were considered significant, printed in bold

^a^*ESI *electrospray ionization, positive or negative mode, *HILIC* hydrophilic interaction chromatography, *m/z* mass-to-charge ratio, *RP* reversed-phase, *RT* retention time

^b^Some metabolites could be categorized as alkylresorcinols (A) [12, 49], betaines (B) [52], microbial metabolites (M), or other metabolites previously [12, 13] associated with wholegrains (O)

^c^Level of identification refers to the reporting guidelines by Metabolomics Standard Initiatives [41]. Only level I (metabolites with matching *m/z*, RT, and MS2 spectra with reference compounds ran with the same procedure) and II (matching *m/z* and MS2 spectra as publicly available spectral libraries) were reported in this table. The full lists of metabolites found identified in DC and later confirmed in RC were reported in Supplementary Tables 1 and 3, respectively

Some metabolites were selected only by either RF or correlation analysis. Valine betaine, AR C23:1-glucuronide, dihydroxybenzoic acid, indolepropionic acid, pyrocatechol sulfate, lysophosphatidylcholine (LPC) (19:0), LPC (22:0), PC (17:0/18:2), glutamine, dihydroactiniolide, pyrrolidone carboxylic acid, gamma-glutamyl-leucine, and two isomers of gamma-glutamyl-valine were selected only by the correlation analysis (Table 2). Conversely, other unknown PCs and lipids were selected only by RF. In addition to the annotated metabolites, several compounds associated with WG remained unidentified (Supplementary Table 1).

After adjustment for confounders, pipecolic acid betaine, tryptophan, hydroxyisoleucine, dimethoxyphenylpropenoic acid, sinapyl alcohol, aminophenol sulfate, tetradecanedioic acid, and three glucuronidated ARs, retained their association (Supplementary Table 1).

### Replication cohort

Among the 61 annotated metabolites from the DC that were also annotated in the RC, 11 were positively correlated with WG intake (FDR < 0.05) (Supplementary Table 3). These were pipecolic acid betaine, tetradecanedioic acid, hydroxyisoleucine, sinapyl alcohol, three glucuronidated ARs (AR C19:0-, C19:1-, and C21:1-glucuronide), 2 PCs [LPC(19:0) and PC(17:0/18:2)], glucuronidated eicosanoid RPneg_511.255@6.50, and an unknown metabolite (HILICneg_177.077@1.29). Among them, pipecolic acid betaine, tetradecanedioic acid, AR C19:0-, C19:1-, and C21:1-glucuronides, HILICneg_177.077@1.29 retained their association after adjustment both in correlation and linear models. Sinapyl alcohol retained its association only after correlation analysis but not in the linear models, and AR C23:1-glucuronide showed an association only after adjustment (Supplementary Table 3). Aminophenol sulfate, dimethoxyphenylpropenoic acid, dihydroxybenzoic acid, and other metabolites that were significant after adjustment in the DC could not be found in RC.

### Microbial metabolites and other wg-related target compounds

In addition to the data-driven approach, we also aimed to replicate compounds previously associated with WG intake or produced by gut microbiota. With this approach, we did not find any additional metabolites related to WG intake (Supplementary Table 4). However, the microbial metabolites indolepropionic acid, dihydroxybenzoic acid isomer, pyrocatechol sulfate, and hippuric acid correlated with WG intake in our data (Supplementary Table 5). Features with matching *m/z* as indoxyl sulfate, indoleacrylic acid, and two isomers of dihydroxyphenylacetic acid (DOPAC) were also associated with WG, but no MS2 data were available to confirm the annotation even after targeted MS2 analysis (Supplementary Table 5). These metabolites, except pyrocatechol sulfate, hippuric acid and a metabolite with matching *m/z* as DOPAC, retained their association after adjustment for confounders (Supplementary Table 1). However, when focusing on the RC, many of the microbial metabolites could not be found in the data, and those that were annotated, e.g., indolepropionic acid and hippuric acid, were not associated with WG intake (Supplementary Table 5).

## Discussion

In this study, we observed associations between WG consumption and the levels of various metabolites in the fasting serum of middle-aged and older men from eastern Finland. Some metabolites, such as pipecolic acid betaine, tetradecanedioic acid, four glucuronidated ARs, and an unknown metabolite, retained their associations in both analyzed cohorts after adjustment for confounders (age, BMI, physical activity, smoking, alcohol, and energy intake).

Pipecolic acid betaine and ARs have been previously associated with WG intake [5, 6, 10, 12]. Pipecolic acid betaine was consistently at the top of the list with a correlation estimate of 0.398 and 0.328 after adjustment in the DC and RC, respectively (Table 2). This finding nominates pipecolic acid betaine as the serum betaine with the strongest association with WG in this study. We also found a consistent association between WG intake and four glucuronidated ARs in this study, with AR C23:1-glucuronide being associated only after adjustment for confounders. Similar to our findings, glucuronidated ARs have previously been reported to associate with WG intake in intervention studies [9, 12]. The odd number of carbon atoms in their side chains highlights the preference of wheat and rye in the study population [8]. However, contrary to previous studies [60–62], we did not find free-form ARs or their metabolites, such as 3-(3,5-dihydroxyphenyl)-propanoic acid and 3,5-dihydroxycinnamic acid [63] in either the DC or the RC, which might be due to differences in the analytical methods and sample preparation techniques. WG intake was found to be associated with dihydroxybenzoic acid (Supplementary Table 5), but the position of the hydroxy groups needs to be confirmed with a reference compound.

Tetradecanedioic acid has previously been extracted from brown rice [64]. Because brown rice was not commonly consumed in Finland in the 80s, this finding may strengthen the previously found association between WG intake and dicarboxylic acids [56], though they have not gained much attention. Sinapyl alcohol constitutes lignin complex in the cereal bran [65] and has been reported to increase after a WG intervention [13]. In this study, it was associated with WG intake in both cohorts after partial correlation but only in adjusted linear models in the RC. This finding may showcase how applying several statistical approaches may enable data exploration from different angles. Consequently, to identify the most robust biomarker candidates, we focused our attention on the metabolites with observable associations in both the RF and the correlation-based approaches.

In the DC, WG intake was associated with some amino acids, namely, glutamine, hydroxyisoleucine, tryptophan, and gamma-Glu-Leu and gamma-Glu-Val. Glutamine, dihydroactiniolide, gamma-glutamylated peptide, and PCs lost their association after adjustment for confounders, suggesting that they might not have a direct association with WG intake. Tryptophan and hydroxyisoleucine, however, retained their association after adjustment. Furthermore, microbial derivatives of tryptophan, namely, indolepropionic acid, as well as metabolites with matching *m/z* as indoxyl sulfate and indoleacrylic acid, retained their direct association after adjustment in the DC. Other microbial metabolites, such as dihydroxybenzoic acid, also showed a positive correlation. These associations between WG intake with amino acids and microbial metabolites were in accordance with previous study reporting increased indoleacetic acid after rye consumption [66], which showed how WG consumption may influence an array of metabolic pathways, including protein and microbial metabolism [67].

In the RC, however, tryptophan did not associate with WG and hydroxyisoleucine lost its association after adjustment. Other microbial metabolites were correlated with WG after adjustment in the DC but could not be identified or lost their associations in the RC. This observation could be due to differences in the consumption patterns caused by different selection criteria between the DC (focus on the healthy Nordic diet [30]) and the RC (focus on egg consumption [31]), despite the same dietary assessment instrument. Similarly, we had previously shown how hippuric acid was related to WG intake in a dietary pattern with fatty fish and berries but not when it was enriched with WG alone [9]. Since the gut microbiome had a stronger association with dietary patterns than with individual dietary constituents [68], different consumption patterns could expectedly be reflected in the gut microbiome and, later, in the microbial metabolites. The variation in the levels of gut microbial metabolites hence might hinder their application as dose-dependent exposure biomarkers [69]. Likewise, the LC–MS instruments used to analyze DC and RC samples were different (LC-Orbitrap-MS vs LC–QTOF–MS, respectively). Therefore, the different analytical capabilities to detect, especially the minor compounds, cannot be ruled out. Despite the different analytical platforms, the repeated association of specific metabolites with WG intake in both the DC and the RC may highlight these metabolites as robust potential biomarkers of WG intake. Future replication in other populations, e.g., with both males and females, or of different age groups, would be necessary to further test the robustness of these metabolites. If these metabolites are proven to be robust across various populations, the next step would be to obtain absolute quantification of these metabolites to understand the kinetics, e.g., time- and dose–response, as well as to investigate the stability, reliability, analytical performance, and reproducibility across different laboratories [7] before they can be used as robust biomarkers of WG intake.

This study has several strengths. The reporting bias was minimized by comprehensive dietary recording accompanied by a picture book, household measurements to estimate the portion sizes, and checking by a nutritionist together with the participants. The WG consumption included the WG cereals in mixed dishes and recipes, which increases the accuracy of habitual intake assessment. Another key strength was the application of a robust metabolomics workflow with stringent quality control and compliance to widely accepted reporting guidelines. Replicated metabolite findings in the RC after adjustment for potential confounding increased the probability that covariates did not primarily drive the observed association. Also, the replicated findings based on two analytical platforms further underline their robustness. These findings may provide a basis for follow-up studies to quantify or examine a causal relationship or biological mechanisms.

There are also several limitations. First, the baseline samples in this cohort were collected during the 1980s, which require validation for current diets and food products. Alterations in the serum metabolome may occur with such prolonged storage even under proper storage conditions. However, this would likely affect all groups similarly and contribute to diluting results rather than systematic bias, which may partially explain the lack of associations. The possibility of not finding metabolites that have been completely degraded or decomposition of metabolites to smaller molecules under such a long storage also cannot be ruled out. Dietary intakes were based on a single 4-day food record, so we could not tell apart if the associated metabolites were due to recent or habitual exposure. Third, the effect of processing, such as sourdough fermentation, could not be distinguished in this study, though it may affect the conversion of WG-derived metabolites [50]. The study design did not enable investigating the causality between WG intake and the metabolic profile. Potential confounding from genetic factors was minimized by selecting men from eastern Finland with a common genetic ancestry [70]. However, it may also restrict the generalizability of results to women and other populations, which may nominate other metabolites as potential biomarkers of WG intake due to variations in the blood metabolome. The contribution of WG intake to the blood metabolome could not be separated from other favorable lifestyle factors, e.g., consumption of a healthy Nordic diet rich in root vegetables and berries, or physical activity. Although physical activity has been included as one of the confounders, it might have not fully accounted for the total contribution of physical activity to metabolic profile and its association with WG intake. Similar arguments would be valid also for other covariates we adjusted for, such as age, BMI, smoking, intake of energy, alcohol, as well as those we could not adjusted for, e.g., healthy Nordic diet, either as a dietary pattern or as individual components, which potentially coexist with WG intake. Hence, follow-up studies in other cohorts are required to validate the findings. The application of different LC–MS platforms for discovery and replication cohorts may raise a possibility of different detection capacity between both instruments, which was minimized by focusing on only metabolites appeared in both discovery and replication cohorts. Finally, univariate and multivariate data analysis strategies have different strengths and weaknesses, and which strategy is best suited for biomarker discovery from nontargeted metabolomics data is still yet unclear. Consequently, studies are de facto being performed using either or both strategies. We, therefore, chose to use both random forest followed by linear models and partial correlation under the rationale that both approaches were complementary. Thus, identifying metabolites that appeared using both techniques would provide a robust selection of biomarker candidates in this exploratory study.

## Conclusions

We examined the fasting serum profile of middle-aged and older men in eastern Finland in relation to WG consumption. High consumption of WG was associated with higher levels of previously reported WG phytochemicals, such as pipecolic acid betaine and glucuronidated alkylresorcinols, as well as novel metabolites, such as tetradecanedioic acid and an unknown metabolite. The retained association after adjustment both in the discovery and replication cohorts showed the potential of these metabolites to reflect WG intake independently of adjusted confounders. These metabolites hence showed potential as biomarker candidates of WG intake, which, after repeated validation attempts, may aid in objective assessment of WG intake in future studies. Further investigations are warranted to assess the influence of individual factors, such as dietary patterns, lifestyle, and gut microbiota, on absorption, digestion, metabolism, and excretion of these biomarker candidates and their causal links with the potential benefits of WG on metabolic health.

## Supplementary Information

Below is the link to the electronic supplementary material.

Supplementary file1 (XLSX 89 KB)

Supplementary file2 (DOCX 266 KB)

## Data Availability

Data described in this manuscript will not be made available, because it contains sensitive personal data of the subjects, which cannot be completely anonymized. These data hence fall under General Data Protection Regulation (GDPR), which require restricted access only to authorized personnel with several protection measures. Interest in the access and use of the data is welcomed by submission of a written proposal to Jyrki Virtanen (jyrki.virtanen@uef.fi). R packages notame for metabolomics data preprocessing (https://github.com/antonvsdata/notame) and MUVR used for the Random Forest analysis (https://gitlab.com/CarlBrunius/MUVR) are available and freely accessible. Supplementary information is available online.
