# Supplementary material for: Serum metabolites associated with wholegrain consumption using nontargeted metabolic profiling: a discovery and reproducibility study
Source: Eur J Nutr. 2022 Oct 6;62(2):713–26. doi: 10.1007/s00394-022-03010-x (PMC9941277; doi:10.1007/s00394-022-03010-x)
Supplement: Supplementary file 2 — Supplementary file2 (DOCX 266 KB) [file 394_2022_3010_MOESM2_ESM.docx]

**SUPPLEMENTARY FIGURE**

**Serum metabolites associated with wholegrain consumption using nontargeted metabolic profiling: a discovery and reproducibility study**

Stefania Noerman, Jyrki K Virtanen, Marko Lehtonen, Carl Brunius, and Kati Hanhineva


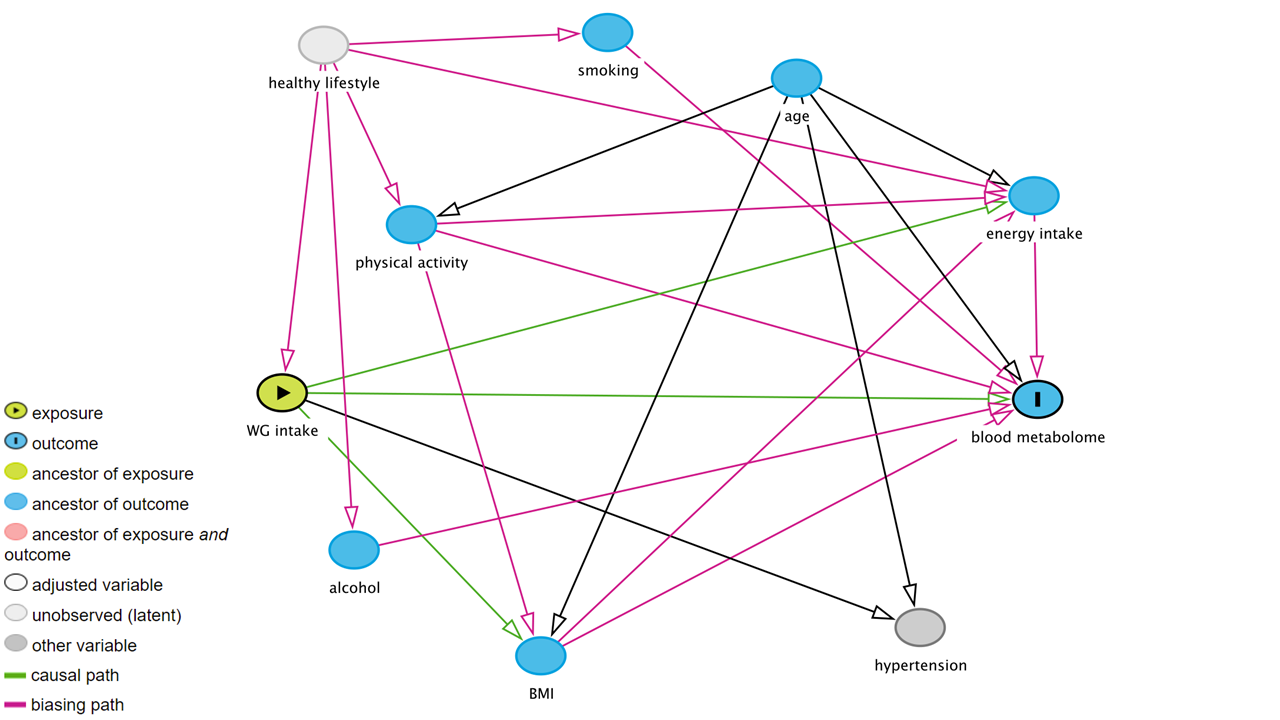


**Supplementary Figure 1**. The relationship among the components was drawn using presumed directed acyclic graph (1). WG intake: wholegrain intake, as the exposure; blood metabolome as the outcome. BMI: body mass index; as other blue oval which were considered as ancestors of outcome. Healthy lifestyle was considered unobserved, though it may affect other observed variables such as physical activity, alcohol consumption, smoking, and energy intake. Green arrows indicate known causal path, while pink arrows indicate biasing path.
